# Upon Certain Mechanical Means of Restoring a Retroverted Uterus

**Published:** 1870-04

**Authors:** H. Webster Jones

**Affiliations:** Accoucheur to Cook County Hospital, Chicago


A MONTHLY RECORD OF

*Medicine, Surgery, and the Collateral Sciences.*

EDITED BY J. ADAMS ALLEN, M.D., LL.D.; AND WALTER HAY, M.D.

VOL. XXVII. — APRIL, 1870. — NO. 4.

### Original Communications.

ARTICLE I. — *Upon Certain Mechanical Means of Restoring a Retroverted Uterus.* By H. WEBSTER JONES, A.M., M.D., Accoucheur to Cook County Hospital, Chicago.

IT has been insisted by the writer, in a previous paper, that every artificial support for the uterus should harmonize with nature's laws; conduce to, rather than hinder, the propagation of species, and be entirely compatible with the bodily and mental comfort of the wearer.

Success depends upon an approximation to these ends, because,

First, Any contrivance which is not based upon exact anatomical knowledge, or which is applied without special investigation of the pathology of the case, is far more likely to do harm than good. And it is due to the lamentable failure of many in these essentials, that the very name of pessary has for years been odious, and that the disorders for the relief of which the instrument was used, have been truly the *opprobria medici*.

Second, Elevators of the womb which *fully* occupy the vagina, and thus hinder the marital relation, are rarely tolerated for a sufficient period to insure even a degree of improvement. Frequent removal and replacement, for any purpose, is oftentimes undesirable, and patients are rarely taught to perform this office for themselves.

Third, A pessary which constantly suggests itself to the wearer, either by its unphilosophical construction, or by its requiring external strapping or bandages for its own support, has the disadvantage of substituting, in its best estate, an artificial and moral anomaly for an inbred and physical evil. The first element of success in dealing with the female organism, is the diversion of the mind from morbid channels, and that physician defeats his own ends who offers his patient a constant reminder of her weakness.

In brief, then, a pessary should possess a *general adaptation* to the anatomy of the parts; a *fitness* to the peculiarities of the case; *no need of frequent removal; non-interference* with coition, and a *self-supporting* and *retentive power*, devoid of bandage or external appliance.

It is a curious study, to view in the light of these principles, the vast array of instruments which have been proposed as uterine supports. If necessity be indeed the mother of art, it is abundantly evident that, for ages, physicians have appreciated both the cause of woman's suffering, and the difficulty of relieving it.

Without attempting a history of these inventions, it is proper to consider them as comprised within four classes:

1st. Those intended for adjustment *anterior* to the *cervix uteri.*

2nd. Those intended to apply power *more directly* to the cervix, by means of a cup or ring embracing it, and dependent upon *exterior* means for their own support.

3rd. The intra-uterine stem pessaries, which act upon the entire uterine cavity, and are supported from without.

4th. The instruments which *elevate* or *retract* the cervix by pressure applied behind it, in the vaginal *cul-de-sac.*

After what has been said, it will be apparent that many and grave objections to the use of most of the pessaries now in vogue, may be properly urged.

All instruments of the *first class* mentioned, are open to the charge of non-conformity to the anatomy of the parts. In order to a cervico-pubic distance of two inches and a half (*vide* JOURNAL, January, 1870,) the globular pessary of Dr. Physick needs a diameter of that extent. This renders its introduction and removal difficult. The colpeurynter of Dr. Garriel is an improvement, but both distend the vagina unnaturally, and rest heavily upon the rectum and urethra, while the latter has a disagreeable appendage in its tube and stop-cock, with regard to the security of which the most courageous patient has many a doubt. Moreover, these instruments are partially obstructive as regards the exit of vaginal and uterine secretions, and totally so as to sexual congress.

Their removal and reposition *by the patient*, for these purposes, is ofttimes equivalent to a nullifying of the objective result, since, in retroversion, they are quite likely to be introduced *under* and *behind* the *cervix uteri*, thus perpetuating the displacement.

Furthermore, they exercise pressure upon a portion of the uterus evidently designed to be especially freed from such contact, and may not be used where inflammation already exists, unless for the relief of a *procidentia*, where they become useful, temporarily, and are the least of two evils.

Pessaries of the *second class* are open to the last objection in a more marked degree. They either embrace the cervix in an inelastic ring, or environ the annule of the *os uteri* with a cup which resists its normal variations.

They must be removed during copulation ; are dependent upon external harness, and as usually constructed, would lift the uterus bodily above the plane of the superior strait.

The " Hoffmann " instrument is of this class, and is in few respects superior to the stuffed rings and discs of the Germans, which have, it is hoped, now become obsolete. There is no " law of suction " whereby, antagonizing the force of gravity, it may elevate itself and the superincumbent organs.

It is properly doubted by authorities of the present day, if indeed intra-uterine stem pessaries, of the *third class*, should ever be used for the purpose of restoring a retroverted or retroflexed uterus.

They attack the organ in its most sensitive tissue, and even if tolerated for a time, are prone to excite early metritic inflammation.

Within the fourth class are included several instruments of fairer fame.

The "lever" of Dr. Cutler, novel in principle, adopts as its fulcrum so sensitive and mobile a structure as the posterior vulval commissure. The writer has found it useful in combating a minor rectocele, but for uterine retroversion, its almost universal result — *perineal irritation* — renders it unavailable.

The flexible ring of Dr. Meigs, based upon a correct idea of pelvic distances, is easily inserted and withdrawn; is in no sense obstructive; demands no external appliance, and is in a remarkable degree unnoticed by the wearer.

But it distends the vagina *laterally*, which is a fault in many cases; tends to obliterate the *reservoir* of the vagina by its tent-like expansion, and does not elevate the *os uteri* in the normal uterine axis. Moreover, its elasticity demands the adoption of a *material* less durable than is consistent with safety in long usage.

The self-extending pessary of Dr. Scattergood is not so flat, and has no objectionable lateral expansion, but its duplex character, and consequent liability to retain the vaginal secretions, make it almost uniformly offensive to the wearer.

In common with these three instruments, the lever pessary of Prof. Hodge possesses the incalculable advantage of avoiding all pressure upon or contact with the *cervix uteri.*

Like the last two, it takes the least sensitive tissues over the inner face of the pubic rami as a *point d'appui.* Unlike them, by its pelvic curve it preserves the receptive and retentive powers of the distal vaginal extremity, and thus avoids sterility. Elevating the *os uteri* above the pelvic floor, it oftentimes *greatly conduces* to the cure of cervical erosions, and by its relief of the torsion of broad ligaments, it sets in motion all the machinery of nature for the cure of hypertrophy of the womb.

It may be worn comfortably during coition, and frequently, by removing an irritable uterus from the possibility of contact, renders this function, for the first time, endurable.

Made of incorrodible material, it has been worn for years, by cleanly persons, without loss of its original smoothness and polish.

Under proper manipulation it may be moulded and adapted strictly to the peculiar demands of the wearer. It bestrides the rectum, instead of resting upon it, and conduces to ease of defecation, and to normal vesical retentiveness.

Being in itself, when in place, a means of determining the patient's need of support, it is certainly the most philosophical, physiological and comfortable pessary yet known.

But however perfect in theory, this support often fails to effect relief, because of its unskillful application, and because of the wearer's ignorance or disregard of certain rules essential.

On the part of the physician, it should be seen to that the instrument selected be neither too wide nor too narrow.

If the former, the pain of introduction will intimidate the operator, and discourage the patient. It is to be remembered, however, that a pessary which passes the *ostium vaginæ* with difficulty, may be worn with entire ease when fairly *in situ*, and particularly is this true of virgins and non-parturient women.

As a general rule, a width over all of more than an inch and three quarters should not be chosen for such cases.

The variable laxity of the tissues concerned makes this far from absolute, but it is certain that *when introduced*, the instrument should never create great lateral tension. Neither should it be too narrow, for thus it loses the intimate lateral contact of the vaginal walls, upon which it is often entirely dependent for the maintenance of an antero-posterior direction. Its retention also depends upon a proper correlation of the instrumental and vaginal width.

Moreover, the pessary should not be so narrow as, in the slightest extent, to compress the *cervix uteri*. In cervical inflammations and hypertrophies this is especially to be guarded against.

When possible, it should always be wide enough to rest at either side of the rectum, when that organ will conduce to maintain rather than overthrow its rectitude.

The length of the instrument, and its height, are important considerations. The former should never exceed three inches and a half, nor the latter be more than two inches (*vide* JOURNAL, January, 1870.)

Contractions of the vagina will often require that these dimensions should only be approximated by a *gradation* of instruments, inserted at proper intervals of time. And in all cases of difficult reposition and adjustment, the condition and form of the

*urethra* must be respected. If it be tender or prominent, the anterior and transverse bar of the lever must be deflected downward and backward.*

Having thus chosen and prepared an instrument, the manner of its insertion is not unimportant. A decubitus upon back or side may be taken by the patient. The position for the use of Dr. Sim's speculum is desirable when difficulty is apprehended. No lubricating material is so good as castor oil, and the operator should remember that the only *distensible* tissues concerned, in the outset, are those of the perineum. Thus he will spare the urethra, and will intrude the pessary obliquely and at one side of that protuberance, avoiding the delicate structure of the *fourchette* as well.

When the uterus is small and movable, it may be possible to pass the distal arch of the instrument behind the cervix, before allowing it to assume a flat position upon the vaginal floor. But, commonly, this latter change should be made so soon as the entire pessary is within the vagina. If the instrument has been well selected, its posterior extremity, by hooking the finger (from underneath) over either longitudinal bar, will be readily sunk until the cervix slips suddenly, and to the great relief of the patient, over the arch, and is suspended, looking downward and backward, in the normal uterine axis.

It is in this last manœuvre, ofttimes, that one finds the patient most resisting, and it is here that the actual reposition of the uterus takes place. If the instrument has been *first* introduced entirely, and it is of a proper length, before the extremity of the cervix may pass over the arch, the uterine axis *must* assume a parallelism with the axis of the superior strait, or even pass into the direction of anteversion. This excess of transit it is

* The writer has abandoned the general use of the "horse-shoe" form of Prof. Hodge's pessary, finding that the "closed bar" lever is more comfortable, and does not hinder coitus, and that the urethral curve above mentioned actually conduces to the propriety of its position.

It does not seem to be understood that any necessary deviation from the commercial form of the pessary may be obtained in a few minutes, by warming *one portion at a time* over the chimney of a kerosene lamp, and fixing it with a *gloved* hand, till the rubber cools and "sets." Too rapid heating results in a loss of polish, which is detrimental.

proper that the physician repeat, by tipping the front cross-bar downward toward the rectum, in order that he may be assured (by simultaneous abdominal palpation) that the *fundus uteri* has left Douglas' *cul-de-sac* to be occupied by the small intestines. With this assurance, and with the knowledge that the whole instrument is adapted to the conformation of the patient, and is, to a certain extent, free to accommodate itself to her position, motion, etc., he may consider that his instant duty is done.

The after-treatment consists in such frequency of observation as the exigency of the case requires; the sensations of the wearer, the condition of the vagina, and the position of the instrument, being the *criteria* whereby the attendant determines the necessity of alterations in the form or dimensions of the pessary, or of a temporary withdrawal of it from over-sensitive tissues.

Daily injections of water (or mucilaginous decoctions, in certain cases,) are essential to cleanliness and health, and it is proper to remark that the various astringents in common use, are liable to fasten upon the instrument the coagulated secretions of the vagina, and demand its occasional withdrawal, lest it become thus roughened and irritating.*

The wide variety of circumstance attending this class of practice, renders any specific rule a mere assumption; but it may be stated, generally, that the need of watchfulness on the part of the physician is proportionate, first, to the *duration* of the displacement.

If of long standing, there may be a degree of vaginal contraction difficult to overcome, or even so great relaxation as will render the mechanical means adopted unstable in its position and office.

Second, the *degree* of retroversion is to be regarded. When this is "complete," the general system may have been wrought up to a very irritable and intolerant state, so that any change is, at best, for a time, of uncertain utility.

* The writer has removed the vulcanized rubber instruments of Prof. Hodge, two and even four years after introduction, to find them perfect in smoothness and polish, and he has found them, within two months, coated with foreign deposits which caused serious annoyance to the parts involved.

Third, the *weight* and *condition* of the uterus may complicate the result; hypertrophies and inflammations will demand the greatest caution, until it be proven that the tendency of these morbid states is toward recovery.

Fourth, *hyperæsthesia* of the vagina and pelvic tissues, generally, will often demand modifications of the form and dimensions of the pessary used, or even require, temporarily, its complete withdrawal.

Again, the failure of the patient to observe and faithfully obey the injunctions of her medical adviser, will often frustrate the plans of the wisest, and bring odium upon the means employed. Instructed that constipation and vesical fullness is her direct foe, she will often suffer the suggestions of nature to pass unheeded, until they become imperative demands, meantime complaining that the support produces pain and impedes motion.

Taught to avoid any abdominal compression, as liable to put upon the pessary more weight than it can sustain, she will often, by tight dressing, or by such labor and exercise as require the forcible contraction of abdominal muscles, annihilate the benefit received. Informed that cleanliness is essential to comfort and health, her daily vaginal ablutions are forgotten, till vaginitis and its attendant evils force her to regard counsel. Besought to note the earliest signs of failure on the part of the instrument to elevate and support the womb, she passes weeks, nay, even months, without such investigation as might provide against the ultimate and grave results of her imprudence.

"What wonder that "*cures* are rare," and "accidents common!"

In brief, under *most favorable circumstances*, and *with watchful care*, it is believed that the pessary of Prof. Hodge will accomplish all that the inventor claims for it, and that it is a safer and better instrument for the relief of lapse, prolapse and retroversion of the uterus, than any mechanical contrivance yet proposed.

Finally, it is due the subject, and the writer's ends, to say that there undoubtedly exists among the profession an inclination to demur at the claims of uterine displacements as an important class of affections.

This fact was met, a few months since, by the publication of a few cases illustrating their bearing upon the female economy, as disturbers of its healthful relations.

It was also true that much of the doubt, silent and expressed, upon the question of the utility of pessaries, was due to false ideas of the anatomy of the pelvic contents. This error was treated very briefly in a more recent paper.

It remained to inform those who had never investigated the subject, of the existence of an instrument which conformed to the anatomical and physiological demands of woman, and to suggest some of the salient points whereon its usefulness might depend.

It is hoped that the medical public may discern, in these attempts, nothing more than an earnest desire to make available to the inquirer the fruits of much experiment, and many and varied disappointments in a wide and interesting field of research.

